# Pulmonary embolism with thrombus trapped in the foramen ovale: a case report

**DOI:** 10.1093/ehjcr/ytag217

**Published:** 2026-03-18

**Authors:** Saki Hosokawa, Kohei Yoneda, Shinobu Hosokawa, Koichi Kishi

**Affiliations:** Department of Cardiology, Tokushima Red Cross Hospital, Tokushima, 103 Irinokuchi, Komatsushima-cho, Komatsushima, Tokushima 773-8502, Japan; Department of Cardiology, Tokushima Red Cross Hospital, Tokushima, 103 Irinokuchi, Komatsushima-cho, Komatsushima, Tokushima 773-8502, Japan; Department of Cardiology, Tokushima Red Cross Hospital, Tokushima, 103 Irinokuchi, Komatsushima-cho, Komatsushima, Tokushima 773-8502, Japan; Department of Cardiology, Tokushima Red Cross Hospital, Tokushima, 103 Irinokuchi, Komatsushima-cho, Komatsushima, Tokushima 773-8502, Japan

## Case description

**Figure 1:**
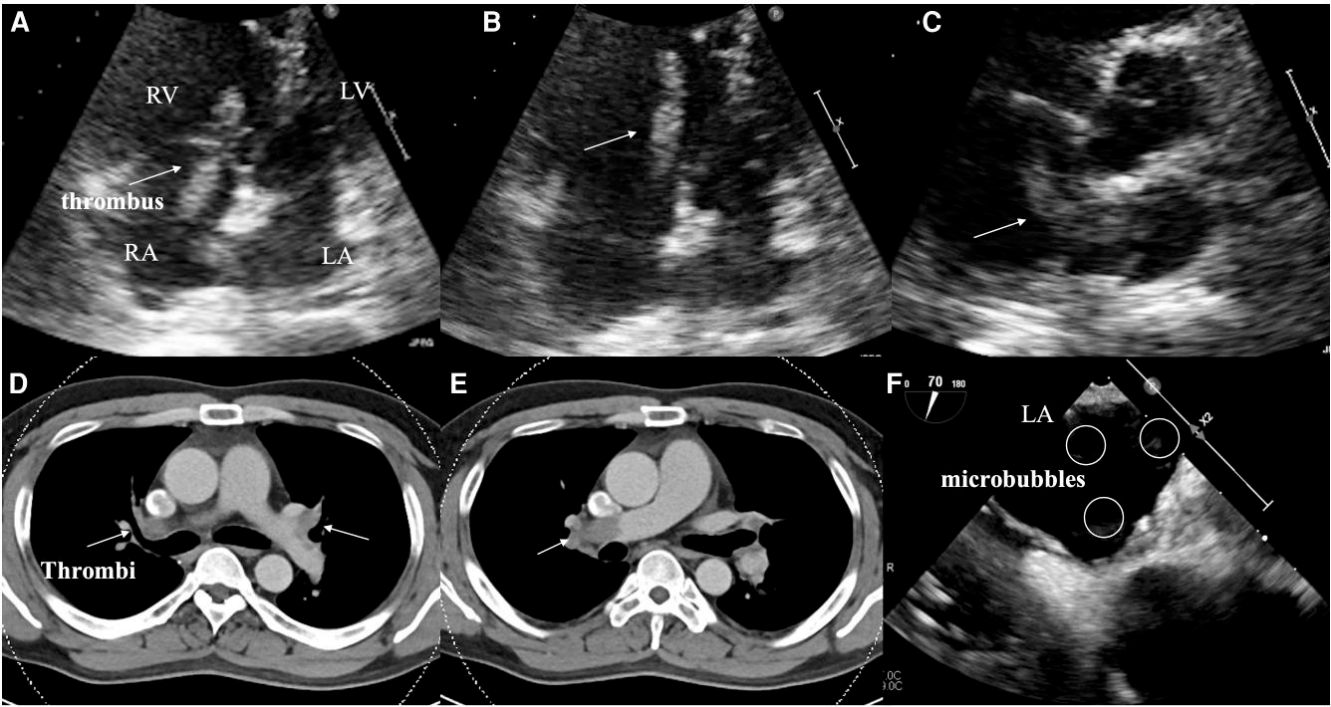
(*A–C*) A mobile, thread-like structure, approximately 4 cm in length, was observed within both atria. During diastole, the structure extended beyond the atrioventricular valves into the ventricles. It was trapped within a patent foramen ovale and positioned between the atria. (*D–E*) Thrombi were identified in both pulmonary arteries on contrast-enhanced computed tomography (CT). (*F*) Transoesophageal echocardiography performed on Day 9 confirmed resolution of the atrial thrombus; however, a bubble test demonstrated grade 2 microbubble passage into the left atrium, even in the absence of a Valsalva manoeuvere.

Owing to progressively worsening dyspnoea, a 39-year-old man was referred to our hospital. His blood pressure was 143/108 mmHg, pulse rate 98 beats/min, and oxygen saturation 98% on 3 L/min of nasal oxygen. A deep vein thrombus was identified on venous ultrasonography of the lower extremities, and thrombi were detected in both pulmonary arteries on contrast-enhanced computed tomography. Mobile thrombi were also observed in both atria, entrapped within a patent foramen ovale (PFO) (*Figure 1*). Pulmonary embolism was diagnosed, and treatment with a direct oral anticoagulant (DOAC; rivaroxaban, 15 mg twice daily for 21 days, followed by 15 mg once daily) was initiated on the same day. As he was haemodynamically stable, thrombolytic therapy was not considered. Transoesophageal echocardiography on Day 9 confirmed resolution of the thrombi in both atria; however, a bubble test demonstrated grade 2 microbubble passage into the left atrium without a Valsalva manoeuvre. Cranial MRI revealed no evidence of cerebral infarction. Although traditional medical management has involved warfarin, several case reports have suggested the efficacy of DOACs.^1^ Moreover, some studies have shown that thrombolytic therapy is associated with significantly increased mortality in haemodynamically stable patients during the early phase of pulmonary embolism, whereas no significant difference in outcomes has been observed between anticoagulation and surgery.^2^ PFO closure was not performed in this patient, as there was no evidence of cerebral infarction on MRI. However, careful consideration should be given to the potential benefits of PFO closure in patients with a history of embolic events.

In this case, the pulmonary embolism and thrombus entrapped within the PFO resolved with oral DOAC therapy, and the patient has remained recurrence free. Although the optimal duration of DOAC therapy in patients at high risk for recurrent pulmonary embolism has not been clearly established, long-term anticoagulation may be necessary in such cases.

## Supplementary Material

ytag217_Supplementary_Data

## Data Availability

All relevant data are included within the article and its Supplementary material.

## Notes

**Consent:** The patient provided written informed consent for publication.
